# The outcome of using intravenous immunoglobulin (IVIG) in critically ill COVID-19 patients’: a retrospective, multi-centric cohort study

**DOI:** 10.1186/s40001-022-00637-8

**Published:** 2022-02-03

**Authors:** Mohammadreza Salehi, Mahdi Barkhori Mehni, Mohammadmehdi Akbarian, Samrand Fattah Ghazi, Nasim Khajavi Rad, Omid Moradi Moghaddam, SaeedReza Jamali Moghaddam, Masoumeh Hosseinzadeh Emam, Sayed Hamidreza Abtahi, Maryam Moradi, Fereshteh Ghiasvand

**Affiliations:** 1 Department of Infectious Diseases, Imam Khomeini Hospital Complex, Tehran University of Medical Sciences, Keshavarz Boulevard, Tehran, Iran; 2 School of Medicine, Tehran University of Medical Sciences, Tehran, Iran; 3 Department of Anesthesiology, Imam Khomeini Hospital Complex, Tehran University of Medical Sciences, Tehran, Iran; 4 Department of Internal Medicine, Imam Khomeini Hospital Complex, Tehran University of Medical Sciences, Tehran, Iran; 5 Trauma and Injury Research Center, Critical Care Medicine Department, Iran University of Medical Sciences, Tehran, Iran; 6 Department of Infectious Disease, School of Medicine, Ziaeian Hospital, Tehran University of Medical Sciences, Tehran, Iran; 7 Department of Anesthesiology, Critical Care Medicine Subspecialty Fellowship, Iran University Medical Sciences, Tehran, Iran; 8 Department of Internal Medicine, School of Medicine, Imam Khomeini Hospital, Tehran University of Medical Sciences, Tehran, Iran; 9 School of Medicine, Iran University of Medical Sciences, Tehran, Iran

**Keywords:** COVID-19, Intravenous immunoglobulin, IVIG, Pneumonia, Coronavirus, Pandemic

## Abstract

**Background:**

During the COVID-19 pandemic, different treatments have been used in critically ill patients. Using intravenous immunoglobulin (IVIG) has been suggested in various studies as an effective option. Our study aims to access the efficacy of IVIG in critically ill COVID-19 patients.

**Methods:**

In this retrospective matched cohort study, records of three tertiary centers with a large number of COVID-19 admissions were evaluated and used. Based on treatment options, patients were divided into two groups, standard COVID-19 treatment (109 patients) and IVIG treatment (74 patients) patients. Also, the effect of IVIG in different dosages was evaluated. Patients with IVIG treatment were divided into three groups of low (0.25 gr/kg), medium (0.5 gr/kg), and high (1 gr/kg) dose. Data analysis was performed using an independent *t* test and one-way analysis of variance (ANOVA) to compare the outcomes between two groups, including duration of hospitalization, intensive care unit (ICU) length of stay, and mortality rate.

**Results:**

The duration of hospitalization in the IVIG group was significantly longer than standard treatment (13.74 days vs. 11.10 days, *p* < 0.05). There was no significant difference between the two groups in ICU length of stay, the number of intubated patients, and duration of mechanical ventilation (*p* > 0.05). Also, initial outcomes in IVIG subgroups were compared separately with the standard treatment group. The results indicated that only the duration of hospitalization in the IVIG subgroup with medium dose is significantly longer than the standard treatment group (*p* < 0.01).

**Conclusion:**

Our data indicate that the use of IVIG in critically ill COVID-19 patients could not be beneficial, based on no remarkable differences in duration of hospitalization, ICU length of stay, duration of mechanical ventilation, and even mortality rate.

## Introduction

Novel coronavirus disease 2019 (COVID-19) pandemic has become a global concern since 2019. By August 2021 more than 4,300,000 people have been sacrificed by this disease [1].

No definitive treatment has been found, so it makes sense to consider the effects of possible treatments on the disease. One of these possible treatments is intravenous immunoglobulin (IVIG), which has been reported to have contradictory effects in previous studies [2, 3].

The IVIG is a plasma-derived drug that was first used to treat some immune deficiencies [4]. It has been used as an adjunct therapy in the management of some infections. Among viral infections, this treatment has been used to prevent and treat various diseases such as influenza, viral hepatitis, rabies, measles, and severe acute respiratory syndrome (SARS) [5]. Studies have revealed that IVIG can be effective in the treatment of severe acute viral pneumonia such as influenza and para-influenza pneumonia [6, 7]. Antigen clearance and immune system modulation are increased by IVIG [8]. Due to the mechanism, safety, and efficacy of this treatment in previous viral infections, some centers have utilized it in severe cases of COVID-19 infection. Despite its efficacy, this treatment is expensive and has various side effects [9]. Thus, the effectiveness and safety of this intervention in COVID-19 infection are debatable. Based on several studies, IVIG administration in COVID-19 patients is controversial [9, 10].

In this study, we intend to investigate the efficacy of adding IVIG to the standard regimen of COVID-19 infection in outcome measures such as duration of hospitalization, mechanical ventilation, and intensive care unit (ICU) length of stay and mortality rate.

## Materials and methods

This retrospective study was conducted on COVID-19 patients in Imam Khomeini hospital complex, Rasul-e Akram, and Ziaeian hospitals in Tehran, Iran.

### Participants

In this study, a total of 202 patients who were admitted between February 2020 and December 2020 were reviewed. These patients were treated with standard methods or IVIG added to standard care. Inclusion criteria were COVID-19 confirmation with real-time polymerase chain reaction (PCR), significant pulmonary findings compatible with radiographic imaging, and critically ill patients in ICU.

Critically ill patients were those with less than 90% oxygen saturation level with a non-rebreather mask, those who needed noninvasive ventilation (NIV) or intubation. Exclusion criteria were age under 18 years, pregnancy, patients with incomplete data, non-ICU patients, those who participated in other clinical trials or received nonstandard treatment due to national or regional protocol, and those who received less than 5 g of IVIG.

### Study arms and treatment plans

**Fig. 1:**
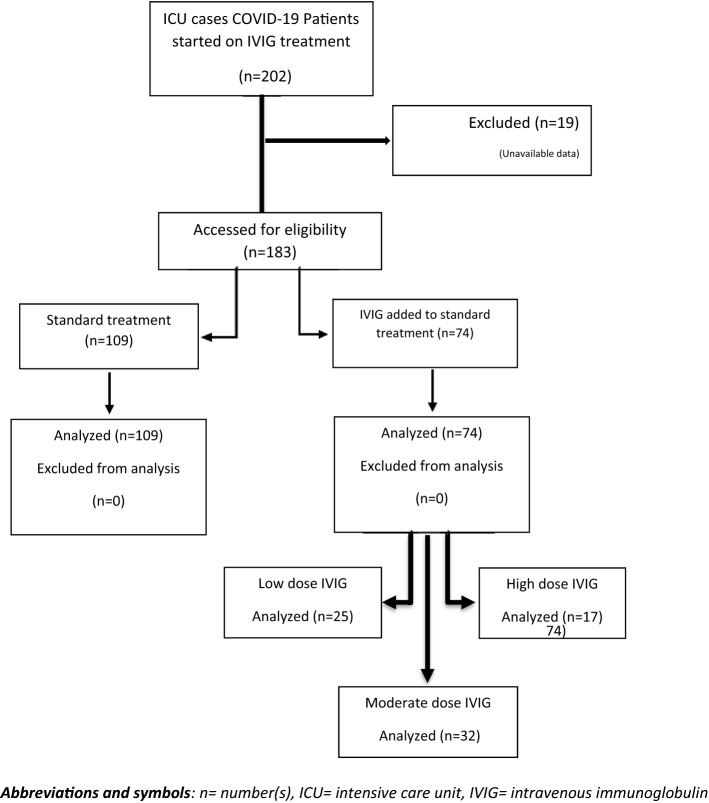
IVIG and standard group assignment

After carefully reviewing the COVID-19 patients’ records based on the hospital information database, and finally considering the inclusion and exclusion criteria, 183 patients were divided into two groups; 109 patients have received standard treatment and 74 patients have received IVIG in addition to the standard treatment (Fig. 1).

In this study, epidemiological, demographic, clinical, laboratory data, management, and outcomes of patients were obtained. Finally, the outcomes including mortality rate, duration of hospitalization, mechanical ventilation, and ICU length of stay were analyzed and compared between two groups.

The standard treatment group regimen was oral hydroxychloroquine (HCQ) 400 mg daily for 5 days plus atazanavir/ritonavir (300/100) daily for 10 days. IVIG treatment was divided into three groups of the low, medium, and high doses (0.25, 0.5, and 1 gr/kg) during 3–5 consecutive days in addition to standard care. Every vial of IVIG with trade name Intratect was liquid dosage form, 5% concentration, and its volume was 100 ml.

### Ethical considerations

The study was approved by the Institutional Review Board of Tehran University of Medical Sciences and exempted from written informed consent [IR.TUMS.VCR.REC.1399.390].

### Measurements and statistical analysis

Distribution of age, sex, initial clinical symptoms, and on admission vital signs were compared between the two groups. Mortality rate, duration of hospitalization, mechanical ventilation, and ICU length of stay were also evaluated.

The results were analyzed using SPSS v.25.0 software. Chi-square tests were performed to evaluate the difference in qualitative data. Shapiro–Wilk test was performed to assess the normality of data distribution. To compare the differences in the quantitative variables of groups, the independent *t* test and one-way analysis of variance (ANOVA) were carried out. Values with *p* < 0.05 were considered statistically significant.

## Results

Based on the inclusion and exclusion criteria, 183 ICU-admitted COVID-19 patients were included in the study, of which 109 (59.6%) were males and 74 (40.4%) were females, with an average age of 62.71 (± 15.46) years. The patients were divided between standard treatment (comprising 109 patients) and IVIG treatment groups (comprising 74 patients) based on their treatment method.

### Clinical and laboratory findings

Baseline clinical characteristics and risk factors of COVID-19 patients (case and control group) are shown in Table 1. Based on the results there were no significant differences between the two groups in demographic data, vital signs on admission, clinical features, laboratory tests, and even risk factors (*p* > 0.05).

**Table 1:** Baseline clinical characteristics and risk factors of COVID-19 patients treated with either IVIG or with standard COVID-19 therapy

Feature	Control group	IVIG group	Statistics	*p* value
Patient number, *N* (%)	109 (59.6)	74 (40.4)		
Male/female, *N* (%)	63.45	45.29		
Age years, mean (STD)	63.28 (16.81)	61.89 (13.38)	0.62	0.536
Days interval from symptoms onset and therapy starting, days No	7.14	7.08	− 0.085	0.933
Vital signs on admission				
*T*	37.33	37.48	0.938	0.394
SO_2_	82.35	80.56	− 0.864	0.389
BP	129.63	127.20	− 0.785	0.434
RR	26.68	26.63	− 0.022	0.983
PR	95.06	96.94	0.647	0.518
Risk factors				
Diabetes	28	29	3.232	0.072
Hypertension	43	35	0.965	0.326
Obesity	5	5	0.105	0.746
Pregnancy	2	0	0.192	0.661
Vasculitis	0	0	–	–
Embolism	0	1	0.041	0.840
Asthma	3	2	0.000	1.000
Bronchiectasis	1	2	0.124	1.000
Member link??	1	0	0.000	0.725
Chemotherapy	0	0	–	–
Corticosteroid therapy	0	0	–	–
Gout	1	1	0.000	1.000
Hemodialysis	4	3	0.000	1.000
Malignancy	9	1	2.77	0.096
Stent	5	5	0.120	0.729
Heart failure	6	10	2.710	0.100
Kidney failure	8	4	0.036	0.849
Liver failure	3	1	0.012	0.914

*N*: number; %: percentage; *T*: temperature; SpO_2_: oxygen saturation; BP: blood pressure; RR: respiratory rate; PR: pulse rate; sounds; PCT: procalcitonin; PCO_2_: partial pressure of carbon dioxide; PH: pulmonary hypertension; Pro-BNP: B-type natriuretic peptide

### Primary and secondary outcome

Primary outcomes including mortality rate, duration of hospitalization, ICU length of stay, and duration of mechanical ventilation were compared in the two groups (Table 2).

**Table 2:** Comparison of primary outcome measures in two groups of patients treated with IVIG and standard treatment group

Feature	Standard group	IVIG group	Statistics	*p* value
No. of patients (%)	109 (59.6)	74 (40.4)	–	–
ICU length of stay (days)	7.33	9.46	1.848	0.066
Duration of hospitalization (days)	11.10	13.74	2.060	0.041
Duration of mechanical ventilation (days)	4.14	4.23	0.119	0.905
Mortality (%)	48 (44.03)	42 (56.75)	2.367	0.124

No: number(s); ICU: intensive care unit; IVIG: intravenous immunoglobulin

According to the primary outcomes, the duration of hospitalization was longer in the IVIG group (*p* < 0.05). The two groups were not significantly different in ICU length of stay, the number of intubated patients, duration of mechanical ventilation, and mortality rate (*p* > 0.05).

Patients in the IVIG treatment group were divided into three groups; low, medium, and high doses of IVIG (0.25, 0.5, and 1 gr/kg). Accordingly, 33.8% of the cases received a low dose, 43.2% medium dose, and 23% high doses (Table 3). In these groups, mortality rate, duration of hospitalization, ICU length of stay, and duration of mechanical ventilation were compared using analysis of variance (ANOVA) for continuous scores and Chi-square (*χ*^2^) for stratified scores. The LSD post hoc test was also used to determine the location of the discrepancy. ANOVA test results are listed in Table 3 along with the results which are significantly different between the two groups.

**Table 3:** Comparison of initial outcome measures in three subgroups of patients treated with IVIG with different doses

Feature	Low-dose IVIG	Medium dose IVIG	High-dose IVIG	*p* value
Number of patients (%)	25 (33.8)	32 (43.2)	17 (23)	
ICU length of stay (days)	10.24	9.77	7.94	0.642
Duration of hospitalization (days)	13.12	16.09	10.39	0.050
Duration of mechanical ventilation (days)	24	29	17	0.703
Mortality rate (%)	13 (52)	18 (56.25)	11 (64.70)	0.83

ICU: intensive care unit; IVIG: intravenous immunoglobulin

There are no significant differences among the three groups of IVIG treatment in primary outcomes as indicated in Table 3 (*p* ≥ 0.05). The comparison between IVIG groups and standard treatment is summarized in Table 4. Based on the results, the duration of hospitalization was longer in the medium-dose IVIG group (*p* < 0.01).

**Table 4:** Comparison of primary outcome measures in three groups of patients treated with IVIG with the standard treatment group

Feature	Low-dose IVIG group and standard group				Medium-dose IVIG group and standard group				High-dose IVIG group and standard group			
	Standard group	Low IVIG	*t* test	*p* value	Standard group	Medium IVIG	*t* test	*p* value	Standard group	High IVIG	*t* test	*p* value
Number of patients	109	25			109	32			109	17		
ICU length of stay (days)	7.33	10.24	− 1.724	0.087	7.33	9.77	− 1.614	− 1.109	7.33	7.94	0.353	0.724
Duration of hospitalization (days)	11.10	13.12	− 1.031	0.305	11.10	16.09	12.887	0.005	11.10	10.39	0.336	0.737
Mortality rate	48	13	0.248	0.618	48	18	1.033	0.310	48	11	1.189	0.275

ICU: intensive care unit, IVIG: intravenous immunoglobulin

Finally, 165 patients were divided into five subgroups based on intubation as shown in Table 5. In this part of the analysis, we need some data of patients such as time of intubation and the exact time of receiving IVIG. Therefore, because of lack of data, 18 out of 183 patients were excluded.

**Table 5:** Mortality rate between five subgroups

Groups	Total (patients)	Mortality (patients)	Discharged (patients)	Mortality (%)
IVIG treatment without intubation	31	0	31	0
IVIG treatment after intubation	29	29	0	100
Intubation after IVIG treatment	8	7	1	87
Standard care without intubation	48	0	48	0
Standard care with intubation	49	36	13	73
Total	165	72	93	44

IVIG: Intravenous Immunoglobulin

The mortality rate was not significantly different between IVIG and standard treatment groups in both intubated and non-intubated subgroups. The mortality rate in subgroups one and four was 0% and the result of the *χ*^2^ test between subgroups three and five showed no statistical difference (*p *value = 0.731 and *χ*^2^ = 0.393).

## Discussion

There have been different studies so far with different results about the effect of IVIG on COVID-19 patients. But clear results have not been obtained [2, 3, 11]. Hence, in this study, the therapeutic effects of IVIG on the confirmed COVID-19 cases were examined and the previous valuable results of related articles were reviewed.

In our retrospective matched cohort study, we examined 183 patients with severe COVID-19 infection who were admitted to the ICU. Seventy-four (40.4%) patients were included in the case group and received IVIG in addition to the standard treatment, but 109 (59.6%) patients in the control group received only standard treatment. On admission, vital signs, clinical signs, laboratory data, and risk factors did not differ significantly between the two groups. Primary outcomes in our study were duration of hospitalization, ICU length of stay, duration of mechanical ventilation, and mortality rate. The results showed that the duration of hospitalization in the IVIG group (13.74 days) was significantly longer than the standard treatment group (11.10 days) (*p *value = 0.041). But there were no significant differences between the other primary outcomes (*p* > 0.05). In our study, we also found that the duration of hospitalization in the medium-dose subgroup of IVIG was longer than the control group and no positive effects were obtained on the duration of mechanical ventilation and mortality of patients.

Based on a randomized clinical trial on 84 patients, 52 patients received a treatment regimen including hydroxychloroquine, lopinavir/ritonavir, and supportive care, plus 400 mg/kg IVIG daily for 3 days, but 32 patients received the same regimen without IVIG. The addition of IVIG to the standard treatment of critically ill COVID-19 patients could not decrease the duration of hospitalization, mechanical ventilation, or mortality rate. In this study, Tabarsi et al. found that the duration of hospitalization was significantly longer in the IVIG treatment group [2]. Many studies have shown that IVIG administration reduces mortality in patients with COVID-19, but increases hospitalization time instead of standard COVID-19 treatment [12, 13].

In a multicenter retrospective cohort study on 325 patients (174 patients in the case group who received IVIG and 151 patients in the control group), 28-day and 60-day mortality were the primary outcomes. Subgroup analysis showed that 28-day mortality in patients with critical type was improved compared to the control group and in these critically ill patients, IVIG reduced the inflammatory response and improved some organ functions, but the length of hospital stay and overall duration of the disease were increased [11].

In another study, IVIG administration in the first 48 h of hospitalization reduced mortality, length of stay, length of stay in the ICU, and duration of mechanical ventilation compared to IVIG administration after 48 h of hospital admission [9]. Herth et al. retrospectively evaluated the clinical courses of 12 COVID-19 patients who received IVIG at various stages of their disease, including within the first 72 h of clinical presentation, after initiation of mechanical ventilation, and after prolonged ventilation and ICU stay. Patients in this study received 0.2 or 0.5 g/kg/day of IVIG for 1 to 4 days. Early IVIG administration in the viral phase of COVID-19 infection can decrease the duration of hospitalization, ICU length of stay, duration of mechanical ventilation, and even mortality rate rather than late administration [9, 14, 15].

Other studies have indicated that using IVIG could reduce the mortality rate, risk of disease progression, and increase survival in critical subgroups of COVID-19 patients [16, 17].

Another question that was examined in this study was the impact of the time, dose, and period of IVIG prescription on its effectiveness. For this purpose, patients with IVIG treatment were divided into three subgroups of low, medium, and high doses. Then the primary outcomes of mortality rate, duration of hospitalization, ICU length of stay, and mechanical ventilation were compared between these subgroups, but no significant differences were obtained. Also, initial outcomes were compared separately with the standard treatment group. The results indicated that only the duration of hospitalization in the IVIG subgroup with medium dose is significantly longer than the standard group, and in other cases, these differences were non-significant. These findings were consistent with the results of Tabarsi et al. [2]. Therefore, in this study, it can be concluded that the use of the IVIG method in COVID-19 treatment is not preferable to its standard treatment.

Difficulty in patient matching and the different timing in IVIG administration were the limitations in our study which should be overcome by conducting RCT studies with a large statistical population.

## Conclusion

Our data indicate that the use of IVIG in critically ill COVID-19 patients could not be beneficial, based on no remarkable differences in duration of hospitalization, ICU length of stay, duration of mechanical ventilation, and even mortality rate.

## Data Availability

Not applicable.

## References

[1] 2021. https://www.worldometers.info/coronavirus/.

[2] Tabarsi P, Barati S, Jamaati H, Haseli S, Marjani M, Moniri A (2021). Evaluating the effects of intravenous immunoglobulin (IVIg) on the management of severe COVID-19 cases: a randomized controlled trial. Int Immunopharmacol.

[3] Mohtadi N, Ghaysouri A, Shirazi S, Shafiee E, Bastani E, Kokhazadeh T (2020). Recovery of severely ill COVID-19 patients by intravenous immunoglobulin (IVIG) treatment: a case series. Virology.

[4] Kaveri S, Maddur M, Hegde P, Lacroix-Desmazes S, Bayry J (2011). Intravenous immunoglobulins in immunodeficiencies: more than mere replacement therapy. Clin Exp Immunol.

[5] Ferrara G, Zumla A, Maeurer M (2012). Intravenous immunoglobulin (IVIg) for refractory and difficult-to-treat infections. Am J Med.

[6] Liu X, Cao W, Li T (2020). High-dose intravenous immunoglobulins in the treatment of severe acute viral pneumonia: the known mechanisms and clinical effects. Front Immunol.

[7] Kanjilal S, Mina MJ (2019). Passive immunity for the treatment of influenza: quality not quantity. Lancet Respir Med.

[8] Nimmerjahn F, Ravetch JV (2008). Anti-inflammatory actions of intravenous immunoglobulin. Annu Rev Immunol.

[9] Xie Y, Cao S, Dong H, Li Q, Chen E, Zhang W (2020). Effect of regular intravenous immunoglobulin therapy on prognosis of severe pneumonia in patients with COVID-19. J Infect.

[10] Liu J, Chen Y, Li R, Wu Z, Xu Q, Li Z (2021). Intravenous immunoglobulin treatment for patients with severe COVID-19: a retrospective multi-center study. Clin Microbiol Infect.

[11] Shao Z, Feng Y, Zhong L, Xie Q, Lei M, Liu Z (2020). Clinical efficacy of intravenous immunoglobulin therapy in critical ill patients with COVID-19: a multicenter retrospective cohort study. Clin Transl Immunol.

[12] Farrokhpour M, Rezaie N, Moradi N, Rad FG, Izadi S, Azimi M (2021). Infliximab and intravenous Gammaglobulin in hospitalized severe COVID-19 patients in intensive care unit. Arch Iran Med.

[13] Gharebaghi N, Nejadrahim R, Mousavi SJ, Sadat-Ebrahimi S-R, Hajizadeh R (2020). The use of intravenous immunoglobulin gamma for the treatment of severe coronavirus disease 2019: a randomized placebo-controlled double-blind clinical trial. BMC Infect Dis.

[14] Herth FJ, Sakoulas G, Haddad F (2020). Use of intravenous immunoglobulin (Prevagen or Octagam) for the treatment of COVID-19: retrospective case series. Respiration.

[15] Pourahmad R, Moazzami B, Rezaei N (2020). Efficacy of plasmapheresis and immunoglobulin replacement therapy (IVIG) on patients with COVID-19. SN Compr Clin Med.

[16] Ali S, Uddin SM, Shalim E, Sayeed MA, Anjum F, Saleem F (2021). Hyperimmune anti-COVID-19 IVIG (C-IVIG) treatment in severe and critical COVID-19 patients: a phase I/II randomized control trial. EClinicalMedicine.

[17] Xiang H-R, Cheng X, Li Y, Luo W-W, Zhang Q-Z, Peng W-X (2021). Efficacy of IVIG (intravenous immunoglobulin) for corona virus disease 2019 (COVID-19): a meta-analysis. Int Immunopharmacol.

